# Supplementary material for: Deep learning-enabled segmentation of ambiguous bioimages with deepflash2
Source: Nat Commun. 2023 Mar 27;14:1679. doi: 10.1038/s41467-023-36960-9 (PMC10043282; doi:10.1038/s41467-023-36960-9)
Supplement: Supplementary file 1 — Supplementary Information [file 41467_2023_36960_MOESM1_ESM.pdf]

Supplementary Information - Deep learning-enabled segmentation of ambiguous bioimages with deepflash2

Matthias Griebel[1*], Dennis Segebarth[2], Nikolai Stein[1], Nina Schukraft[2], Philip Tovote[2,3], Robert Blum[4], and Christoph M. Flath[1*]

[1]Department of Business and Economics, University of Würzburg, Germany
[2]Institute of Clinical Neurobiology, University Hospital Würzburg, Germany
[3]Center for Mental Health, University Hospital Würzburg, Germany
[4]Department of Neurology, University Hospital Würzburg, Germany

[*]Corresponding authors {matthias.griebel, christoph.flath(at)uni-wuerzburg.de}

S1 Supplementary Note - Partly out-of-distribution data

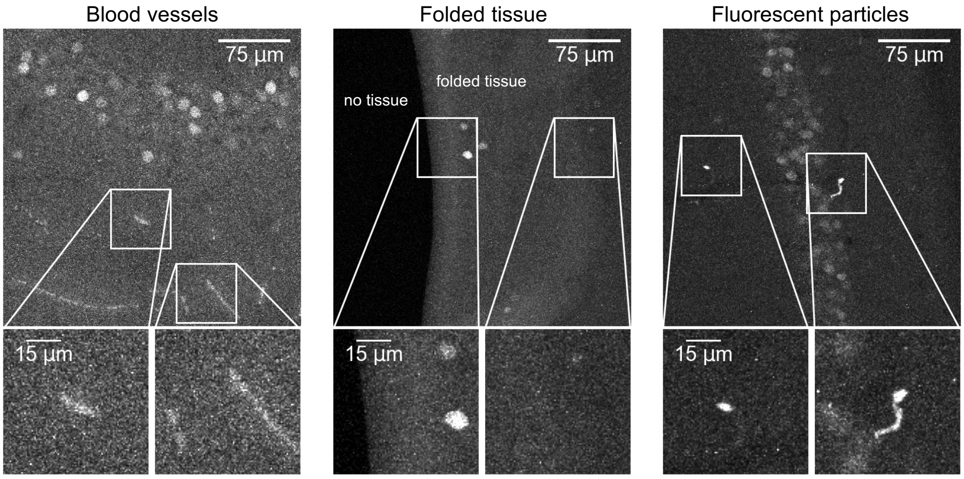

Figure S1.1: **Partly out-of-distribution images.** Image crops and zoom-ins of three error categories in the extended *cFOS in HC* dataset: blood vessels, if the images contained blood vessels; folded tissue; fluorescent particles, if there was at least one strongly fluorescent particle, unrelated to the actual fluorescent label.

S2 Supplementary Note - Extended performance comparison

S2.1 Expert annotation comparison

We utilize the inter-expert variation as a proxy for data ambiguity. Fig. S2.1 depicts this variation by means of similarity scores between expert segmentations and estimated GT (derived via simultaneous truth and performance level estimation (STAPLE) [1]). We use the dice score (DS, first row) for semantic segmentation and the mean Average Precision (mAP, second row) for instance segmentation. Both metrics exhibit a clear correlation for all experts. The performance of the experts on the train and test data is also approximately equally distributed, except for *PV in HC* dataset. The Average Precision at different IoU-thresholds η (third row) reveals further differences between the experts. For the *mScarlet in PAG* dataset, for instance, Expert 3 has a comparatively low AP at low η, but outperforms the other experts at high η. In other words, Expert 3 has a low detection performance $AP_{IoU=0.50}$ but the segmentations of the detected instances are very precise.

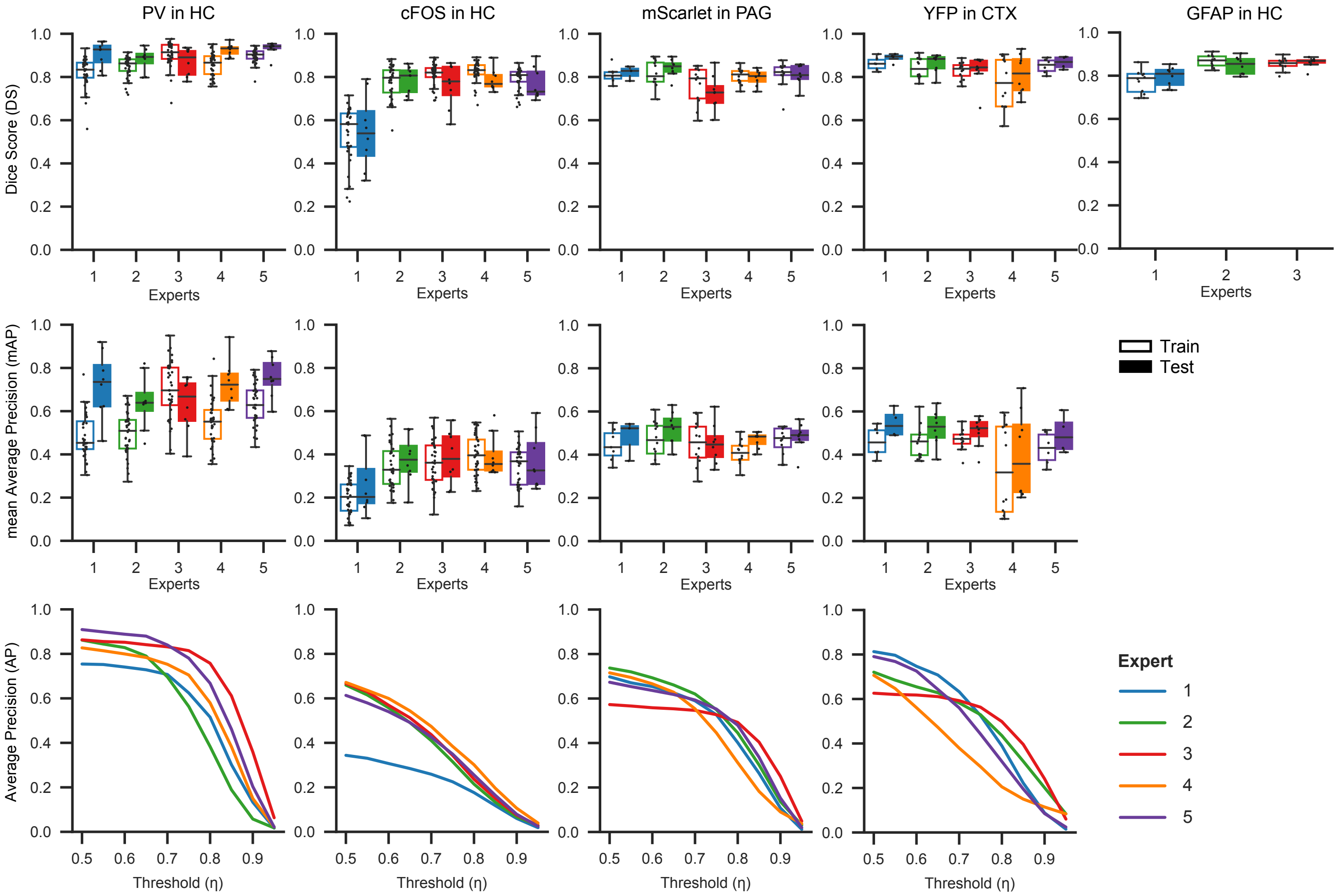

Figure S2.1: **Expert annotation performance.** The expert annotation performance is measured by the experts' annotation similarity to the estimated ground truth (STAPLE [1]). For semantic segmentation (first row) and instance segmentation (second row) the train sets ($N = 36$ images for *PV in HC* and *cFOS in HC*; $N = 12$ for the remaining datasets) and test sets ($N = 8$ images for each dataset) are depicted separately. The lines in the third row depict the mean over the Average Precision (AP) at different thresholds for all data points (train and test set combined, $N = 44$ images for *PV in HC* and *cFOS in HC*; $N = 20$ for the remaining datasets). The datasets are annotated by different experts (expert IDs cannot be used as identifiers across datasets). Box plots are defined as follows: the box extends from the first quartile (lower bound of the box) to the third quartile (upper bound of the box) of the data, with a center line at the median. The whiskers extend from the box by at most 1.5x the interquartile range and are drawn down to the lowest and up to the highest data point that falls within this distance.

S2.2 Method performance comparison

To ensure that our results are robust and reproducible we repeat our experiments with different seeds. This changes the train-validation splits and weight initialization for each repetition. In addition to the out-of-the-box *cellpose** approach [2] we include fine-tuned *cellpose* models and ensembles in this comparison. We train the *cellpose* models via five-fold cross-validation with the default training settings from the command line interface (500 epochs, 0.2 learning rate, batch size of 8). The resulting *cellpose* ensemble consists of five models similar to the deepflash2 model ensembles. As the *cellpose* command line interface does not implement training via cross-validation, we also include models trained on a single train-validation-split into our analysis. These models are simply selected from the trained model ensembles. The results of three experiment repetitions for all methods are reported in Table S2.1.

Table S2.1: **Method performance comparison on immunofluorescence datasets.** Average predictive performance measured by similarity to the estimated ground truth (STAPLE [1]) on the hold-out test sets (N = 8 images for each dataset) over three repetitions. The deepflash2 models were either completely initialized from a truncated normal distribution (rand) or with *imagenet*-pretrained weights in the encoder (pre). *indicates methods that were not fine-tuned on the respective dataset). Source data are provided as a Source Data file.

	PV in HC	cFOS in HC	mScarlet in PAG	YFP in CTX	GFAP in HC
Method	Semantic Segmentation - Mean DS (std. deviation)				
Otsu*	0.101 (–)	0.033 (–)	0.156 (–)	0.743 (–)	0.600 (–)
U-Net (2019)	0.863 (0.010)	0.769 (0.010)	0.756 (0.008)	0.850 (0.037)	0.762 (0.015)
nnunet	0.891 (0.002)	0.797 (0.000)	0.821 (0.002)	0.883 (0.000)	0.799 (0.000)
deepflash2 (rand)	0.873 (0.006)	0.779 (0.001)	0.759 (0.003)	0.874 (0.000)	0.798 (0.001)
deepflash2 (pre)	**0.910** (0.010)	**0.822** (0.002)	**0.830** (0.000)	**0.884** (0.002)	**0.817** (0.001)
	Instance Segmentation - Mean mAP (std. deviation)				
cellpose*	0.541 (–)	0.268 (–)	0.148 (–)	0.304 (–)	– (–)
cellpose (single)	0.610 (0.012)	0.329 (0.010)	0.415 (0.004)	0.499 (0.014)	– (–)
cellpose (ensemble)	0.628 (0.028)	0.350 (0.004)	0.432 (0.002)	0.511 (0.010)	– (–)
U-Net (2019)	0.548 (0.024)	0.305 (0.016)	0.337 (0.003)	0.455 (0.059)	– (–)
nnunet	0.643 (0.004)	0.368 (0.002)	0.443 (0.002)	0.527 (0.003)	– (–)
deepflash2 (rand)	0.606 (0.008)	0.342 (0.004)	0.344 (0.004)	0.502 (0.006)	– (–)
deepflash2 (pre)	**0.689** (0.020)	**0.412** (0.003)	**0.469** (0.003)	**0.536** (0.006)	– (–)
	Detection - $AP_{IoU=0.50}$ (std. deviation)				
cellpose*	0.701 (–)	0.404 (–)	0.237 (–)	0.536 (–)	– (–)
cellpose (single)	0.844 (0.025)	0.662 (0.008)	0.666 (0.011)	0.805 (0.027)	– (–)
cellpose (ensemble)	0.851 (0.031)	0.688 (0.015)	0.686 (0.009)	0.823 (0.018)	– (–)
U-Net (2019)	0.844 (0.016)	0.566 (0.017)	0.573 (0.004)	0.755 (0.044)	– (–)
nnunet	0.825 (0.003)	0.647 (0.011)	0.670 (0.003)	0.807 (0.005)	– (–)
deepflash2 (rand)	0.811 (0.005)	0.612 (0.007)	0.585 (0.005)	0.794 (0.008)	– (–)
deepflash2 (pre)	**0.857** (0.021)	**0.697** (0.007)	**0.706** (0.004)	**0.801** (0.011)	– (–)

deepflash2 outperforms the other methods for both semantic and instance segmentation on all datasets. Moreover, the results show that the ensemble-based methods *nnunet* and deepflash2 yield very stable results (low std. deviations) across all datasets, while the U-Net of [3], based on a single model, is subject to higher performance variability.

We also report the results of the detection task, which is commonly measured by the $AP_{IoU=0.50}$. In contrast to the mAP that provides a measure for the quality of the segmentation, the $AP_{IoU=0.50}$ metric measures the "counting" performance of a method. That is, for instance, whether the same cell is annotated or not. The *cellpose* ensemble performs on par with the deepflash2 model on the detection task on the *YFP in CTX* dataset.

A more detailed analysis in Fig. S2.2 reveals that the fine-tuned *cellpose* models yield similar results to deepflash2 at low IoU-thresholds η but constantly perform worse for higher thresholds.

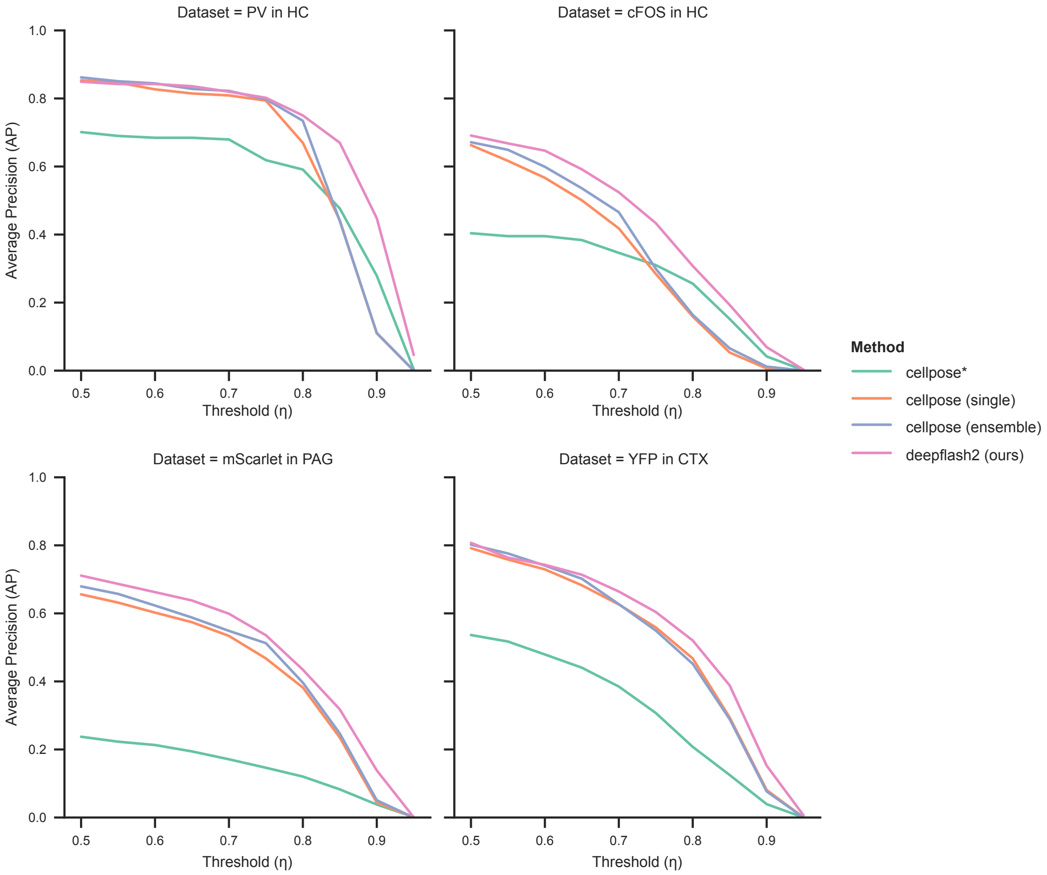

Figure S2.2: **deepflash2 vs. *cellpose*** The lines depict the instance segmentation performance using the mean of the Average Precision ($N = 8$ hold-out test images for each dataset) at a certain IoU-threshold η over 3 repetitions. The out-of-the-box model ensembles *cellpose** do not achieve competitive performance. The fine-tuned *cellpose* models (single and ensemble) yield similar results to deepflash2 at low η but constantly perform worse at higher η. Source data are provided as a Source Data file.

Table S2.2 reports the detailed results on the challenge datasets *gleason*, *monuseg*, and *conic*. We used the same default training hyperparameter settings for all datasets except for the *gleason* dataset, where we changed one hyperparameter to increase the receptive field of the image tiles (zoom-out factor of 4) to account for the large tumor regions.

Table S2.2: **Method performance comparison on challenge datasets.** Average predictive performance measured by similarity to the (estimated) ground truth on the test sets (*gleason*: $N = 49$, *monuseg*: $N = 15$, *conic*: $N = 15$) over three repetitions. The *average* column contains the arithmetic mean of the different class scores. The deepflash2 models were initialized with *imagenet*-pretrained weights in the encoder. Source data are provided as a Source Data file.

	gleason - semantic segmentation (average DS)						
Method	benign	grade 3	grade 4	grade 5			average
nnunet	0.905	0.535	0.532	**0.084**			0.662
deepflash2 (pre)	**0.911**	**0.646**	**0.618**	0.000			**0.753**
	monuseg - instance segmentation (average mAP)						
	nucleus						average
nnunet	0.333						0.333
deepflash2 (pre)	**0.375**						**0.375**
	conic - instance segmentation (average mAP)						
	epithel.	lymph.	plasma	eosinoph.	neutroph.	con. tissue	average
nnunet	0.056	0.253	0.430	**0.250**	0.112	0.328	0.255
deepflash2 (pre)	**0.062**	**0.353**	**0.436**	0.241	**0.116**	**0.341**	**0.274**

S3 Supplementary Note - Exploration of model ensembling strategies

During the development of deepflash2, we explored various approaches for modeling the different experts, for example, by modeling each expert with a single head (output) as suggested by [4]. The final deepflash2 implementation, however, builds upon the findings of Segebarth et al. [5]. They explore (i) single models, each trained on one of the annotators, (ii) a model trained on the consensus annotations, and (iii) an ensemble of models trained on the consensus annotations. Strategy (iii) delivers the most accurate and reliable results and represents the suggested use for deepflash2. Another simple baseline would be an ensemble of single models, each trained on one of the annotators (similar to i) except that the expert models are not evaluated individually but used as an ensemble). Figure S3.1 shows that the ensemble of models trained on the consensus annotations will typically outperform the merging strategy.

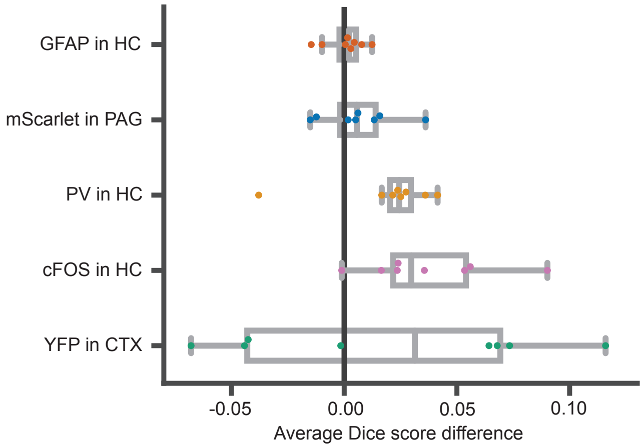

Figure S3.1: **Expert model ensembles vs. STAPLE ensembles.** Performance difference between an ensemble of single models, each trained on one of the annotators, and an ensemble of models trained on the STAPLE consensus annotations. The points show the average difference in the Dice score to the est. ground truth on a single test image ($N = 8$ for each dataset) over three repetitions. Values < 0 indicate a higher Dice score for the first strategy, and values > 0 have a higher Dice score for the second strategy. Box plots are defined as follows: the box extends from the first quartile (lower bound of the box) to the third quartile (upper bound of the box) of the data, with a center line at the median. The whiskers extend from the box by at most 1.5x the interquartile range and are drawn down to the lowest and up to the highest data point that falls within this distance.

S4 Supplementary Note - Performance comparison of annotation strategies and most efficient use of expert time

The annotation strategy choice needs to trade-off the number of training images, which should represent the diversity of the data, against the annotation quality gains from multiple annotations. To explore the relative advantage of these competing factors we simulated two different annotation strategies. The first strategy (STAPLE) required the annotation of all images by all available experts with subsequent GT estimation using STAPLE [1], which is our default strategy. The second strategy (DIFFERENT) required the experts to annotate different images, resulting in much larger training sets. The results of the trained model ensembles were evaluated on the est. GT of the hold-out test sets, considering the annotations of all available experts. The results (Fig. S4.1) indicate that the DIFFERENT strategy is superior when only a few image annotations are available. In this case, the model performance benefits from more (but less precise) image-annotation-pairs to capture the diverse data distribution. The STAPLE strategy is superior when more training images are available. Here, the data distribution is sufficiently represented in the smaller training set with more precise annotations.

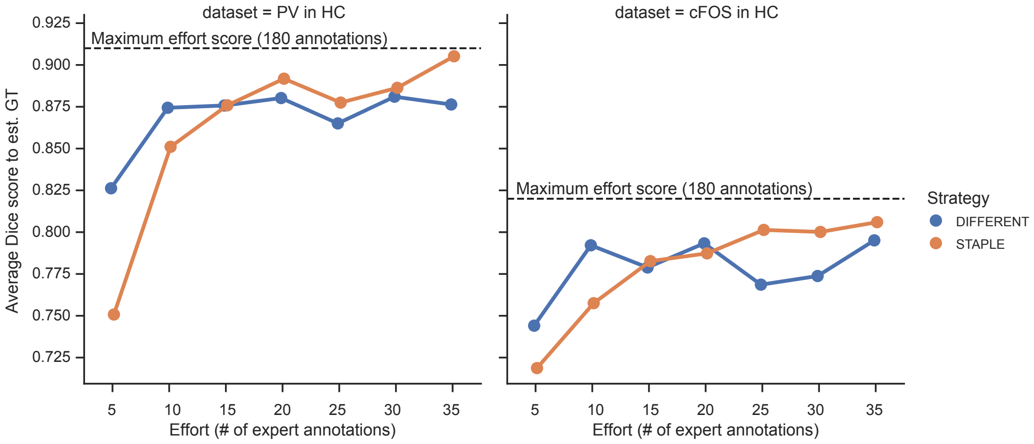

Figure S4.1: **Analysis of different annotation strategies.** Semantic segmentation performance on the hold-out test sets ($N = 32$, 8 images for each dataset). The STAPLE strategy requires annotating that all training images are annotated by all available experts (five) with subsequent GT estimation using STAPLE (e.g., the annotation effort of five results in a single training image-annotation-pair, the annotation effort of 35 results in seven training image-annotation-pairs). The DIFFERENT strategy requires the experts to annotate different images, resulting in larger training sets (the number of training images equals the number of expert annotations). The experiments were simulated by sampling from all available training images and expert annotations and were repeated three times. The maximum effort score indicates the score when the models are trained on all available training data (36 images with annotations from five experts each) using the STAPLE strategy.

S5 Supplementary Note - Aleatoric and epistemic uncertainty

deepflash2 generates *predictive* uncertainty maps that allow the quantification of data ambiguities and simplify the verification of the predictions. We show that these uncertainties can be related to the agreement of the human experts (see Fig. 2e in the main paper). The *predictive* uncertainty is composed of the *aleatoric* and *epistemic* uncertainty (see Methods). Examples of the different uncertainty types are depicted in Fig. S5.1. The *aleatoric* (data or per-measurement) uncertainty can be derived from the predicted probabilities. It is low for probabilities close to zero or one, and high for probabilities around 0.5. This results, for instance, in high *aleatoric* uncertainties for the border regions of the segmented cell nuclei or somata. In contrast, *epistemic* (model) uncertainty foremost captures the uncertainty of planar areas that are entirely ambiguous. In these areas, the models' predictions may differ considerably as a clear distinction between the foreground and background classes is not feasible.

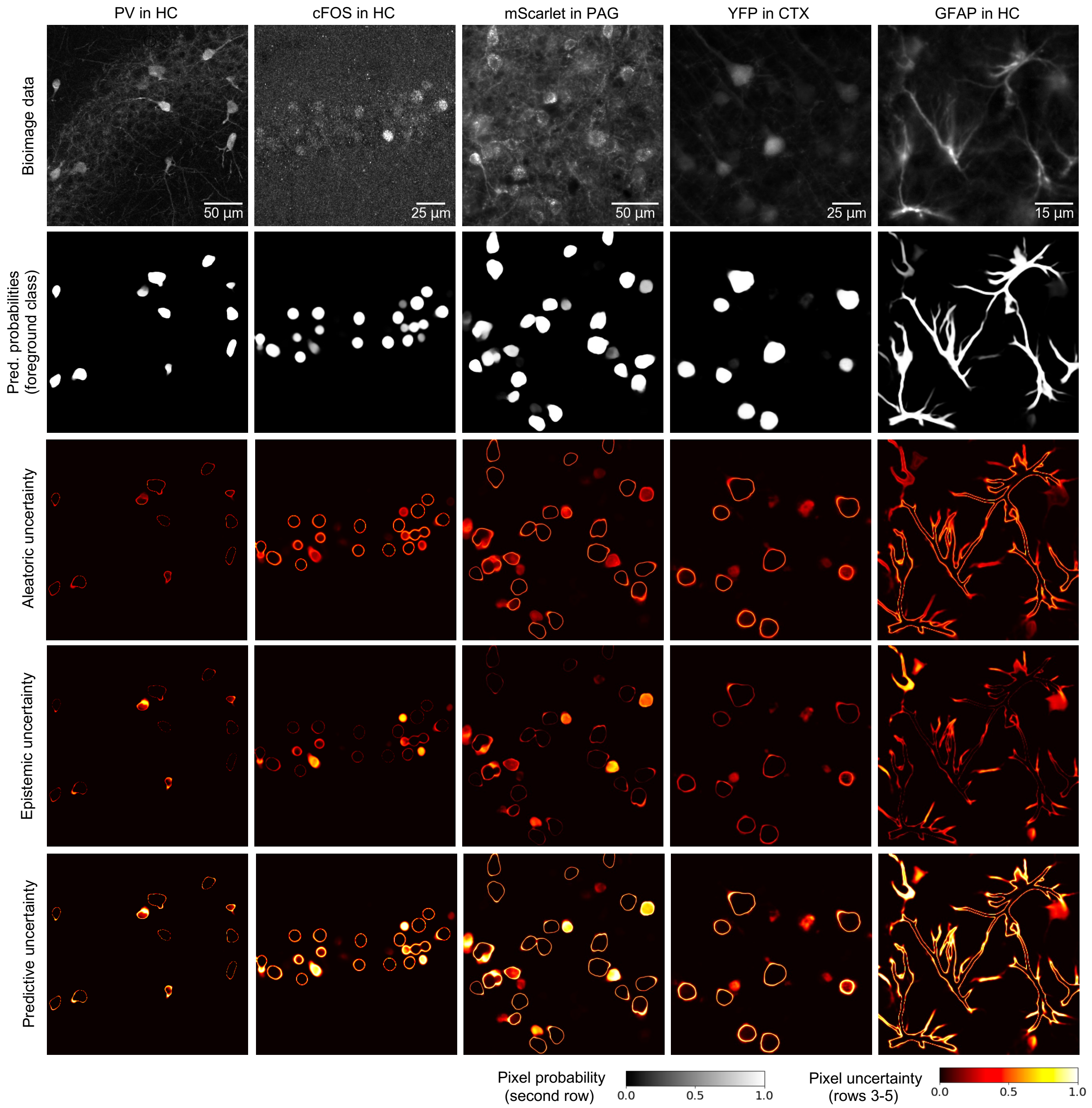

Figure S5.1: **Visualization of uncertainty types.** Representative image sections from the test sets of five immunofluorescence imaging datasets (first row) and corresponding ensemble probability map $p(\mathbf{y} = k \mid \mathbf{X})$ for the foreground class k (second row). The *aleatoric* (third row) and *epistemic* (fourth row) uncertainty maps are combined into the *predictive* uncertainty map $Var_{p(\mathbf{y}|\mathbf{X},\theta)}$ (fifth row). *Aleatoric* uncertainties foremost emerge in the border regions of the segmented cell nuclei or somata. High *epistemic* uncertainties typically occur in planar areas where a clear distinction between the foreground and background classes is not feasible. deepflash2 computes the *predictive* uncertainty by default.

S6 Supplementary Note - Extended out-of-distribution detection analysis

The quality assurance process of deepflash2 (Section 2.5 in the main paper) helps the user prioritize the review of more ambiguous images and also facilitates the detection of out-of-distribution images. Such images differ from the training data and are typically prone to erroneous predictions. In our exemplary *cFOS in HC* dataset for out-of-distribution detection we differentiate between fully out-of-distribution images, e.g., images that visualize different immunofluorescent labels, and partly out-of-distribution images. The latter exhibit the same properties as the training data but also contain previously unseen structures such as blood vessels, folded tissue, or fluorescent particles (see Section S1 and Fig. S1.1). Fig. S6.1 shows that the uncertainty scores U (Equation 5 in Section 2.2 in the main paper) of the partly out-of-distribution images are significantly higher than the uncertainty scores of the in-distribution images (No error). However, the error categories are not distributed evenly. On the one hand, blood vessels and folded tissue images cover a high and relatively wide range of uncertainty scores. On the other hand, fluorescent particle images exhibit uncertainty scores close to the median of the in-distribution images. A possible explanation is the small proportion of unseen structures (a single strongly fluorescent particle unrelated to the actual fluorescent label) in these images. Using the proposed heuristic search strategy (Fig. 2f in the main paper) such images would be detected at a later stage, however, the inclusion of such images into the bioimage analysis would possibly not impair the results.

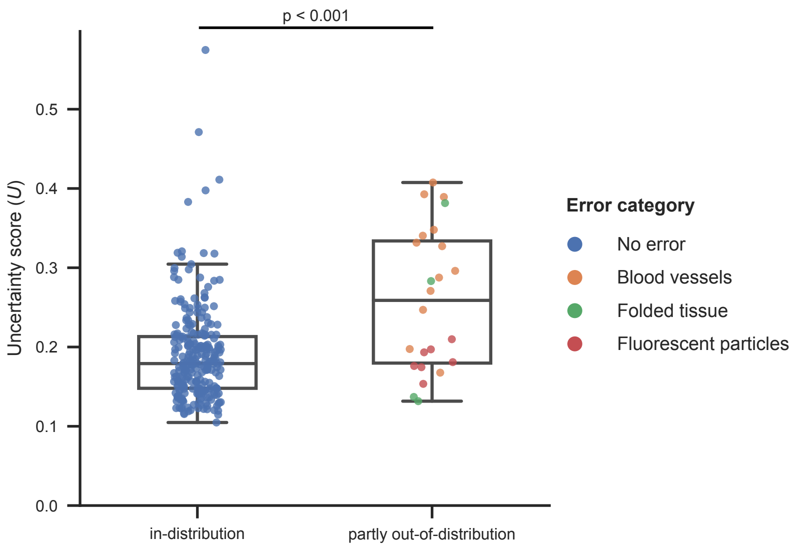

Figure S6.1: **Uncertainty scores and out-of-distribution error categories.** Uncertainty score comparison for the *cFOS in HC* out-of-distribution dataset. The dataset comprises in-distribution (No error, $N = 264$) and partly out-of-distribution images (blood vessels ($N = 13$), folded tissue ($N = 4$), fluorescent particles ($N = 7$)). The p-value results from a two-sided non-parametric Mann–Whitney U test. Partly out-of-distribution images typically exhibit a higher uncertainty score. Box plots are defined as follows: the box extends from the first quartile (lower bound of the box) to the third quartile (upper bound of the box) of the data, with a center line at the median. The whiskers extend from the box by at most 1.5x the interquartile range and are drawn down to the lowest and up to the highest data point that falls within this distance. Source data are provided as a Source Data file.

References

[1] Warfield, S. K., Zou, K. H. & Wells, W. M. Simultaneous truth and performance level estimation (staple): an algorithm for the validation of image segmentation. *IEEE transactions on medical imaging* **23**, 903–921 (2004).

[2] Stringer, C., Wang, T., Michaelos, M. & Pachitariu, M. Cellpose: a generalist algorithm for cellular segmentation. *Nature Methods* **18**, 100–106 (2021).

[3] Falk, T. *et al.* U-net: deep learning for cell counting, detection, and morphometry. *Nature Methods* **16**, 67–70 (2019).

[4] Guan, M., Gulshan, V., Dai, A. & Hinton, G. Who said what: Modeling individual labelers improves classification. In *Proceedings of the AAAI Conference on Artificial Intelligence*, vol. 32 (2018).

[5] Segebarth, D. *et al.* On the objectivity, reliability, and validity of deep learning enabled bioimage analyses. *eLife* **9**, e59780 (2020).
