# Supplementary material for: Deep learning-enabled segmentation of ambiguous bioimages with deepflash2
Source: Nat Commun. 2023 Mar 27;14:1679. doi: 10.1038/s41467-023-36960-9 (PMC10043282; doi:10.1038/s41467-023-36960-9)
Supplement: Supplementary file 2 — Reporting Summary [file 41467_2023_36960_MOESM2_ESM.pdf]

nature portfolio

Corresponding author(s): Matthias Griebel, Christoph M. Flath

Last updated by author(s): Feb 20, 2023

Reporting Summary

Nature Portfolio wishes to improve the reproducibility of the work that we publish. This form provides structure for consistency and transparency in reporting. For further information on Nature Portfolio policies, see our Editorial Policies and the Editorial Policy Checklist.

Statistics

For all statistical analyses, confirm that the following items are present in the figure legend, table legend, main text, or Methods section.

n/a	Confirmed	
[ ]	[x]	The exact sample size (*n*) for each experimental group/condition, given as a discrete number and unit of measurement
[ ]	[x]	A statement on whether measurements were taken from distinct samples or whether the same sample was measured repeatedly
[ ]	[x]	The statistical test(s) used AND whether they are one- or two-sided<br>*Only common tests should be described solely by name; describe more complex techniques in the Methods section.*
[x]	[ ]	A description of all covariates tested
[x]	[ ]	A description of any assumptions or corrections, such as tests of normality and adjustment for multiple comparisons
[ ]	[x]	A full description of the statistical parameters including central tendency (e.g. means) or other basic estimates (e.g. regression coefficient) AND variation (e.g. standard deviation) or associated estimates of uncertainty (e.g. confidence intervals)
[ ]	[x]	For null hypothesis testing, the test statistic (e.g. *F*, *t*, *r*) with confidence intervals, effect sizes, degrees of freedom and *P* value noted<br>*Give P values as exact values whenever suitable.*
[x]	[ ]	For Bayesian analysis, information on the choice of priors and Markov chain Monte Carlo settings
[x]	[ ]	For hierarchical and complex designs, identification of the appropriate level for tests and full reporting of outcomes
[ ]	[x]	Estimates of effect sizes (e.g. Cohen's *d*, Pearson's *r*), indicating how they were calculated

Our web collection on statistics for biologists contains articles on many of the points above.

Software and code

Policy information about availability of computer code

Data collection	Image acquisition was performed using a Zeiss Axio Zoom.V16 microscope, equipped with a Zeiss HXP 200C light source, an Axiocam 506 mono camera, and an APO Z 1.5x/0.37 FWD 30mm objective.
Data analysis	Data annotation was performed using ImageJ v2.3/Fiji v.1.53. The deepflash2 code (https://github.com/matjesg/deepflash2) uses Python 3 with the numpy v1.21.6, scipy v1.7.3, opencv v4.5.4, SimpleITK v2.0.2, ipywidgets v7.7.1, pytorch v1.13.1, fastai v2.7.11, timm v0.6.12, albumentations v1.2.1, segmentation-models-pytorch v0.3.0 packages; We compared the segmentation performance to nnunet v1.6.6, cellpose v.6.6 GitHub version with commit hash 316927e (August 26, 2021), and a U-Net implementation (Tensorflow 1.x) of Falk et al. (2019). Statistical analyses were performed using pingouin v0.5.3; Figures were created using seaborn v0.11.2 and matplotlib v3.4.2

For manuscripts utilizing custom algorithms or software that are central to the research but not yet described in published literature, software must be made available to editors and reviewers. We strongly encourage code deposition in a community repository (e.g. GitHub). See the Nature Portfolio guidelines for submitting code & software for further information.

Data

Policy information about availability of data

All manuscripts must include a data availability statement. This statement should provide the following information, where applicable:

- Accession codes, unique identifiers, or web links for publicly available datasets
- A description of any restrictions on data availability
- For clinical datasets or third party data, please ensure that the statement adheres to our policy

All data and trained DL models are available on Zenodo (https://doi.org/10.5281/zenodo.7653312). The data will be moved to a permanent and citable repository upon final acceptance of the paper. The cFOS in HC dataset from Segebarth et al. (2020) is also available on Dryad (https://doi.org/10.5061/dryad.4b8gtht9d). The figure source data is available on GitHub (https://github.com/matjesg/deepflash2/releases/tag/paper_source_data). The external challenge data is available at the respective challenge websites (gleason: https://gleason2019.grand-challenge.org/, monuseg: https://monuseg.grand-challenge.org/, conic: https://conic-challenge.grand-challenge.org/ based on the Lizard data set)

Human research participants

Policy information about studies involving human research participants and Sex and Gender in Research.

Reporting on sex and gender	*Use the terms sex (biological attribute) and gender (shaped by social and cultural circumstances) carefully in order to avoid confusing both terms. Indicate if findings apply to only one sex or gender; describe whether sex and gender were considered in study design whether sex and/or gender was determined based on self-reporting or assigned and methods used. Provide in the source data disaggregated sex and gender data where this information has been collected, and consent has been obtained for sharing of individual-level data; provide overall numbers in this Reporting Summary. Please state if this information has not been collected. Report sex- and gender-based analyses where performed, justify reasons for lack of sex- and gender-based analysis.*
Population characteristics	*Describe the covariate-relevant population characteristics of the human research participants (e.g. age, genotypic information, past and current diagnosis and treatment categories). If you filled out the behavioural & social sciences study design questions and have nothing to add here, write "See above."*
Recruitment	*Describe how participants were recruited. Outline any potential self-selection bias or other biases that may be present and how these are likely to impact results.*
Ethics oversight	*Identify the organization(s) that approved the study protocol.*

Note that full information on the approval of the study protocol must also be provided in the manuscript.

Field-specific reporting

Please select the one below that is the best fit for your research. If you are not sure, read the appropriate sections before making your selection.

☒ Life sciences ☐ Behavioural & social sciences ☐ Ecological, evolutionary & environmental sciences

For a reference copy of the document with all sections, see nature.com/documents/nr-reporting-summary-flat.pdf

Life sciences study design

All studies must disclose on these points even when the disclosure is negative.

Sample size	Computational study: sample sizes were determined by the number of available images in the evaluation data sets. If the train and test sets were not defined, we used a random train-test-split of 80/20.
Data exclusions	The images with id 1608 from the dataset cFOS in HC and PV in HC (Segebarth et al. (2020)) were removed from the test sets to ensure a balanced evaluation (eight test images across all five datasets in this study)
Replication	Computational study: This study uses data of mice experiments for evaluation purposes. The paper does not report any biological effects that could be reproduced. However, the computational experiments were independently repeated three times with similar results.
Randomization	The paper does not report any biological effects from life science experiments, so randomization is not applicable. For the computational study random sampling of images into train and test set was performed (see sample size).
Blinding	Computational study: blinding not applicable as this paper does not report any biological effects from the life science experiments. There was nothing the experts could be blinded for. However, during the data annotation process, they did not receive information on the annotations of the other experts.

Reporting for specific materials, systems and methods

We require information from authors about some types of materials, experimental systems and methods used in many studies. Here, indicate whether each material, system or method listed is relevant to your study. If you are not sure if a list item applies to your research, read the appropriate section before selecting a response.

Materials & experimental systems

n/a	Involved in the study
☐	☒ Antibodies
☒	☐ Eukaryotic cell lines
☒	☐ Palaeontology and archaeology
☐	☒ Animals and other organisms
☒	☐ Clinical data
☒	☐ Dual use research of concern

Methods

n/a	Involved in the study
☒	☐ ChIP-seq
☒	☐ Flow cytometry
☒	☐ MRI-based neuroimaging

Antibodies

Antibodies used	rabbit anti-RFP (Biomol, 600-401-379, 1:1,000); rabbit anti-GFAP (Acris, DP014, 1:200); chicken anti-GFP (Abcam, Ab13970, 1:1,000);
Validation	The anti RFP colabels red fluorescent proteins specifically. Anti-GFAP labels typical astrocytes and radial glia in the hippocampus, as expected.<br>Anti-GFP co-labels cells expressing GFP or YFP.

Animals and other research organisms

Policy information about studies involving animals; ARRIVE guidelines recommended for reporting animal research, and Sex and Gender in Research

Laboratory animals	All mice used in this study were bred in the animal facility of the Institute of Clinical Neurobiology, at the University Hospital of Wuerzburg, Germany, and housed under standard conditions with access to food and water ad libidum. VGlut2-IRES-Cre knock-in mice (stock no. 208863), as well as Thy1-YFP mice (stock no. 003782) were obtained from Jackson Laboratory. Additionally, we used wildtype mice with the genetic background C57BL/6J (Charles River, CRL:027). Only male mice at an age between four and eight months were used.
Wild animals	No wild animals were used in the study.
Reporting on sex	Only male mice were used in this study.
Field-collected samples	No field collected samples were used in the study.
Ethics oversight	All experiments and experimental procedures were in accordance with the guidelines set by the European Union and our local veterinary authority (Veterinaeramt der Stadt Wurzburg). In addition, all experiments and experimental procedures were approved by our institutional Animal Care, the Utilization Committee, and the Regierung von Unterfranken, Wurzburg, Germany (License numbers: 55.2–2531.01-95/13 and 55.2.2-352-2-509). ARRIVE guidelines were applied.

Note that full information on the approval of the study protocol must also be provided in the manuscript.
