# Supplementary material for: Deep learning-enabled segmentation of ambiguous bioimages with deepflash2
Source: Nat Commun. 2023 Mar 27;14:1679. doi: 10.1038/s41467-023-36960-9 (PMC10043282; doi:10.1038/s41467-023-36960-9)
Supplement: Supplementary file 3 — Description of Additional Supplementary Files [file 41467_2023_36960_MOESM3_ESM.pdf]

Title: Supplementary Software

Description:

- **deepflash2-0.2.2.tar.gz**: Source code and Readme, see https://github.com/matjesg/deepflash2
- **deepflash2-0.2.2-py3-none-any.whl**: built distribution
